# 35156 Sensory mechanisms of atypical motor variability and regularity in autism spectrum disorder

**DOI:** 10.1017/cts.2021.650

**Published:** 2021-03-31

**Authors:** Robin L. Shafer, Zheng Wang, James Bartolotti, Matthew W. Mosconi

**Affiliations:** 1 University of Kansas; 2 University of Florida

## Abstract

ABSTRACT IMPACT: This project aims to better understand mechanisms of sensory and motor deficits in individuals with ASD with the goal of informing diagnosis and treatment development. OBJECTIVES/GOALS: Over-reliance on both visual and proprioceptive feedback have both been observed during motor behavior in persons with Autism Spectrum Disorders (ASD), suggesting that separate sensory feedback processes may be selectively altered during different behaviors. The objective of this study is to clarify sensory mechanisms of fine motor control in ASD. METHODS/STUDY POPULATION: Participants with ASD (N=43) and controls (N=23) matched on age (10-20 yrs) and non-verbal IQ completed tests of precision gripping. Participants were instructed to press on force sensors with their index finger and thumb so that a moving bar corresponding to their force output reached and stayed as stable as possible at the level of a stationary target bar. Visual feedback was manipulated by changing the visual gain of the force bar (low, medium and high). The force bar moved more per change in force output at higher gains. Proprioceptive feedback was manipulated by applying 80 Hz tendon vibration at the wrist to induce an illusion of muscle contraction. This was compared to a condition with the tendon vibrator turned off. Force variability (standard deviation) and regularity (sample entropy) were examined. RESULTS/ANTICIPATED RESULTS: Controls showed increased force variability with the tendon vibration on compared to off (t = -3.372, p < 0.001); however, the ASD group showed no difference in force variability between the tendon vibration conditions (t = -0.960, p = 0.338). Individuals with ASD had stronger age-associated reductions in force variability relative to controls across tendon vibrator and gain conditions (Group x Age: t = -4.05, p < .001). The ASD group also had greater age-associated increases in force regularity relative to controls, especially at higher gain levels (Group x Gain Level x Age: t = -3.22, p = 0.001). Unlike the ASD group for whom regularity increased with age in both tendon vibration conditions, controls only showed these age-related gains when the tendon vibrator was off (Group x Vibration Frequency x Age: t = 2.46, p = .014). DISCUSSION/SIGNIFICANCE OF FINDINGS: Our findings indicate that while controls integrate proprioceptive and visual feedback online to accurately adjust fine motor behavior, persons with ASD rely mostly on visual feedback. Our results suggest delayed development of sensory integration and reduced reliance on multisensory feedback during online fine motor control in persons with ASD.

